# Dual Antiviral Functions of Antibodies Targeting African Swine Fever Virus p17 Protein: Viral Inhibition and ADCC Induction

**DOI:** 10.3390/v18080841

**Published:** 2026-08-01

**Authors:** Shengmei Chen, Chunhao Jiang, Zhanhao Lu, Jing Lan, Qiang Fu, Yuan Sun, Tao Wang, Hua-Ji Qiu

**Affiliations:** 1 State Key Laboratory of Animal Disease Control and Prevention, Professional Laboratory for African Swine Fever (Harbin), National High Containment Facilities for Animal Diseases Control and Prevention, Harbin Veterinary Research Institute, Chinese Academy of Agricultural Sciences, Harbin 150069, China; 2 College of Life Science and Engineering, Foshan University, Foshan 528231, China

**Keywords:** African swine fever, African swine fever virus, antibody-dependent cellular cytotoxicity, p17

## Abstract

African swine fever virus (ASFV) causes African swine fever (ASF), a highly lethal disease in pigs. Vietnam has approved two ASF live-attenuated vaccines (LAVs), but their efficacy and safety remain controversial, and no reliable, highly effective commercial ASF vaccine is available yet. Humoral immunity plays an important role in protection against ASFV infection. However, there is still controversy regarding whether ASFV infection can induce antibodies with neutralizing activity. Antibody-dependent cellular cytotoxicity (ADCC), as an antibody-mediated protective mechanism, offers a novel perspective for screening protective ASFV antigens. This study evaluated five structural proteins (pCP312R, pA104R, pA151R, p17, and pF317L) as subunit vaccine candidates based on their ability to induce antibodies that inhibit viral replication and mediate ADCC. The recombinant proteins were expressed in *Escherichia coli*, purified, and used to immunize pigs. Immune sera collected two weeks after the third immunization were tested for their ability to inhibit ASFV replication in porcine alveolar macrophages (PAMs) using rASFV-Gluc/EGFP. ADCC activity was assessed using a stable HEK293T-p17 cell line as target cells and porcine peripheral blood mononuclear cells (PBMCs) as effectors, with cytotoxicity measured by lactate dehydrogenase release. All five recombinant proteins were successfully expressed and purified. Immunization with pCP312R, pA104R, p17, and pF317L induced the production of specific antibodies in pigs, but only anti-p17 antibodies significantly inhibited ASFV replication in PAMs. The p17 is highly conserved across different ASFV genotypes and is predicted to contain a transmembrane domain. Anti-p17 antibodies effectively mediated PBMCs to specifically kill target cells, demonstrating significant ADCC activity. Moreover, the HEK293T-p17 cell line was specifically recognized by anti-ASFV sera. These findings indicate that p17 is a dual-functional antigen capable of eliciting antibodies that both inhibit viral replication and mediate ADCC in vitro. Furthermore, we have developed an in vitro platform for screening protective ASFV antibodies based on viral inhibition and ADCC, providing candidate targets for the development of next-generation ASF subunit vaccines.

## 1. Introduction

African swine fever (ASF), caused by African swine fever virus (ASFV), was first reported in Kenya, East Africa, in 1921. It primarily circulated among wild boars, particularly warthogs, which typically show no clinical signs upon infection [1]. In 1957, ASF spread from Africa to Portugal and subsequently caused large outbreaks across parts of Europe and South America. By the 1990s, most regions outside of Sardinia and Africa had successfully eradicated ASF [2,3,4]. However, in 2007, ASFV re-emerged in Georgia and rapidly spread through the Caucasus and Eastern Europe. The same virulent genotype II strain reached Northeast China in 2018, then disseminated throughout East Asia and Southeast Asia [5,6,7]. During this ongoing spread, ASFV has continuously evolved, giving rise to naturally attenuated strains, recombinants strains, and potential immune-escape mutants, posing unprecedented challenges for early diagnosis, surveillance, and vaccine development [8,9,10,11]. Although significant progress has been made in biosecurity-based control measures in recent years, the development of an effective vaccine against ASF remains a major challenge due to its large and complex genome, high environmental stability, and sophisticated immune evasion mechanisms. Vietnam has conditionally approved two live-attenuated vaccines (LAVs) based on the ASFV-G-ΔI177L and ASFV-G-ΔMGF strains [12,13]. However, subsequent studies have raised concerns about their safety, including risks of viral persistence and horizontal transmission [14,15,16]. To date, no safe, efficacious, and commercially viable ASF vaccine is available for widespread use, and the disease control situation remains challenging.

Inactivated vaccines exhibit good safety profiles but fail to elicit effective and protective immunity. LAVs have demonstrated partial protection in experimental settings but are associated with significant safety issues, such as reversion to virulent potential for recombination with circulating field strains and limited efficacy against emerging variant strains [17,18]. In contrast, subunit vaccines, characterized by well-defined components, high safety, and suitability for standardized manufacturing, are emerging as a leading strategy in current ASF vaccine development. However, key bottlenecks persist, primarily the lack of clearly defined protective antigens and insufficient immunogenicity of candidate antigens [19].

ADCC is mediated by the binding of the antibody Fc region to Fc-gamma receptors (Fc*γ*Rs) on the surface of immune effector cells, thereby triggering effector functions that play a crucial role in eliminating virus-infected target cells and preventing further disease progression [20]. This mechanism has been well-documented across multiple pathogens, including human immunodeficiency virus (HIV), simian immunodeficiency virus (SIV), and influenza virus [21,22]. Notably, antibodies induced by different viral antigens exhibit significantly distinct ADCC activity. For instance, immune sera targeting antigens such as p54 and CD2v can mediate target cell lysis rates ranging from 50% to 76%. This activity correlates positively with IFN-γ secretion levels but shows no correlation with total IgG titers [23]. In contrast, antibodies induced by cell lines expressing p30 or pE120R on the cell surface elicit ADCC activity of 19–30% [24,25]. Given the current lack of reliable and universally accepted neutralizing antibodies against ASFV, Fc-mediated effector functions are emerging as a quantifiable metric for evaluating antibody quality and predicting potential protective efficacy. Given the large and complex ASFV genome, which encodes more than 200 proteins, there may exist novel antigens that have not yet been fully explored but are capable of eliciting potent immune responses. Therefore, systematic screening of ASFV antigens with a protective potential is crucial for advancing ASF vaccine development. The five candidate antigens selected in this study were all chosen based on the existing literature, as they have been demonstrated to play key roles in virion assembly, immune modulation, or other critical processes, making them relatively promising targets.

pCP312R exhibits strong immunogenicity and interacts with the host protein RPS27A to suppress host cellular protein function, thereby promoting viral replication [26,27]. The ASFV non-structural protein pA151R possesses thioredoxin-like activity essential for maintaining the redox environment required for viral replication, and its downregulation significantly impairs ASFV replication [28]. pF317L has been shown to bind IKK*β*, inhibit its phosphorylation, block NF-*κ*B activation, and suppress pro-inflammatory cytokine production, thus facilitating viral replication [29]. pA104R localizes to the viral nucleoid, where it binds to the viral DNA and induces bending, contributing to the packaging and stability of the viral genome [30].

The p17 protein, encoded by the *D117L* gene, is one of four minor capsid proteins and is located in the inner capsid layer of the ASFV virion [31]. Loss of p17 function severely impairs the proteolytic processing of pp220 and pp62, disrupting proper virion assembly during infection [32]. p17 is abundantly expressed intracellularly and localizes to the endoplasmic reticulum (ER) and Golgi apparatus, where it participates in regulating cell proliferation, oxidative stress, and ER stress responses [33]. Due to its high conservation and stable expression during infection, p17 has already been adopted as a reliable molecular diagnostic target in ASFV nucleic acid amplification assays [34].

In this study, the five candidate antigens were expressed in *Escherichia coli* and used to immunize pigs to generate antigen-specific antibodies. The resulting antisera were then evaluated in vitro for their ability to inhibit ASFV replication. Antibodies exhibiting antiviral activity were further assessed for their capacity to mediate ADCC, thereby enabling a systematic evaluation of their functional immune characteristics.

## 2. Materials and Methods

### 2.1. Cells, Viral Strains, Antibodies, and Plasmids

HEK293T cells, porcine alveolar macrophages (PAMs), the recombinant virus rASFV-Gluc/EGFP, and the cell-adapted strain ASFV-P121 are all routinely maintained in our laboratory. Anti-ASFV sera and eukaryotic expression plasmids encoding the five candidate antigens (pCP312R, pA151R, pA104R, p17, and pF317L) are also stably preserved in our laboratory. rASFV-Gluc/EGFP was constructed using the highly virulent ASFV strain HLJ/18 as the backbone, with the coding sequences of Gaussia luciferase (Gluc) and enhanced green fluorescent protein (EGFP) inserted downstream of the viral *MGF300-4L* gene to enable co-expression, without deletion of any other viral genes [35]. ASFV-P121 is an attenuated, cell-adapted strain derived by serial passage of an ASFV isolate in HEK293T cells. Its complete genome has been sequenced and shown to be genetically stable [36]. Anti-ASFV sera from the pigs were immunized with ASFV-SY18-ΔCD2v/UK, a *CD2v*/*UK*-deleted ASFV mutant [37], while the negative control sera were obtained from specific-pathogen-free (SPF) pigs.

### 2.2. Design and Construction of Plasmids

Since the transmembrane protein p17 encoded by *D117L* is difficult to express in a soluble form, the transmembrane domain of p17 (residues 38–60) was replaced with a 3×G4S linker, and the modified p17 was then fused with ferritin for expression. A biotechnology company performed codon optimization of the sequences of *CP312R*, *A151R*, *A104R*, *D117L-ferritin*, and *F317L* genes, synthesized them, and cloned them into the prokaryotic expression vectors pCold-I or pET-30a. Given that p17 was expressed as inclusion bodies in the prokaryotic system and remained undetectable in the eukaryotic system, ferritin fusion was employed to facilitate its soluble expression. SpyTag was added to the N-terminus of A104R and CP312R, whereas A151R was tagged with SpyTag at its C-terminus. The C-terminus of F317L contains a 3×G4S linker, a SpyTag, as well as His and Strep tags. The resulting plasmids were designated as pCold-CP312R, pCold-A151R, pET30a-A104R, pET30a-D117L-ferritin, and pET30a-F317L. Upon receipt of the plasmids, PCR identification was carried out using the plasmids as templates, followed by Sanger sequencing validation performed by Harbin Ruiboxingke Biotechnology Co., Ltd., Harbin, China.

### 2.3. Prokaryotic Expression and Purification of Recombinant Proteins

The constructed prokaryotic expression plasmids were transformed into BL21 (DE3) competent cells (Tiangen, Beijing, China) and plated onto LB agar plates containing the appropriate antibiotics, followed by overnight incubation at 37 °C. Single colonies were picked and inoculated into liquid LB medium for shaking of cultures to prepare seed cultures. These were then diluted 1:200 into fresh medium and grown until the OD_600nm_ reached 0.6–0.8. The cultures were then divided into two groups: one induced with IPTG (TaKaRa, Tokyo, Japan) and the other left uninduced as a control, both incubated at 16 °C for induction. After induction, the cells were harvested, resuspended in PBS (pH 7.3), and lysed by sonication on ice. Lysates were centrifuged to separate soluble (supernatant) and insoluble (pellet) fractions, with the uninduced control processed in parallel. Protein expression was initially assessed by SDS-PAGE (Sigma-Aldrich, St. Louis, MO, USA), followed by Coomassie Brilliant Blue staining, and further confirmed by Western blotting: proteins were transferred onto PVDF membranes, blocked, and sequentially probed with a 6 × His primary antibody and a goat anti-mouse fluorescent secondary antibody, followed by washing and detection using an infrared imaging system. For purification, induced cells were lysed by sonication, centrifuged, and filtered; the clarified supernatant was incubated with Ni-NTA resin (Cytiva, Marlborough, MA, USA) overnight at 4 °C. The next day, the mixture was loaded into a Ni^2+^–NTA column and washed to remove impurities, and the target protein was eluted. The eluted fraction was analyzed by SDS-PAGE, concentrated and mixed with glycerol, and stored at −20 °C.

### 2.4. Pig Immunization for Antigen Screening

This study randomly assigned 18 healthy commercial piglets aged 6 weeks into five groups (*n* = 3). Each candidate antigen was emulsified with the commercial veterinary adjuvant Montanide ISA 15A VG at a ratio of 9:1 and injected intramuscularly into the neck at a dose of 100 μg. Two booster immunizations were given at 14 and 28 d post-immunization (dpi) using the same dose and route. The trial concluded at 42 dpi. Serum samples were collected prior to vaccination (0 dpi) and at 42 dpi for subsequent immunological assays. All the piglets were humanely euthanized by a lethal dose of sodium pentobarbital (Bio PIKE, Beijing, China) at 42 dpi. All animal experiments were conducted in strict accordance with ethical guidelines and approved by the Institutional Animal Care and Use Committee of Harbin Veterinary Research Institute, Chinese Academy of Agricultural Sciences, Harbin, China.

### 2.5. Indirect Immunofluorescence Assay (IFA)

HEK293T cells were seeded in 24 well plates (5 × 10^4^ cells/well) and transfected when they reached approximately 80% confluence with 1 μg of each candidate antigen plasmid using PEI in Opti-MEM (Thermo Fisher Scientific, Waltham, MA, USA). After 6–8 h, the transfection mix was replaced with fresh Dulbecco’s modified Eagle’s medium (DMEM) (Gibco, New York, NY, USA). At 36 h post-transfection, the cells were washed with cold PBS, fixed in absolute ethanol at −20 °C for 30 min, and rehydrated with PBS. Primary antibodies included the sera from the immunized pigs at 0 and 42 dpi diluted 1:10 in PBS, mouse anti-Flag antibody (Abclonal, Wuhan, China) diluted 1:200 as the positive control, and negative sera from SPF pigs serving as the negative control. Following primary antibody incubation at 37 °C for 2 h, the cells were washed twice with PBST, FITC-labeled rabbit anti-pig IgG or goat anti-mouse IgG secondary antibodies (Thermo Fisher Scientific, Waltham, MA, USA) were added and incubated at 37 °C in the dark for 1 h. After washing the wells three times with PBST for 3 min per wash, 200 μL of PBS was added to each well, and fluorescence was examined under a fluorescence microscope.

### 2.6. Virus Inhibition Assay

To evaluate the ability of antibodies induced by immunization with five candidate antigens pA151R, pCP312R, pA104R, p17-ferritin, and pF317L to inhibit ASFV replication in vitro, PAMs were seeded at 10^5^ cells per well in 96-well plates and cultured at 37 °C with 5% CO_2_ until adherent. Sera collected from each pig at 0 and 42 dpi were diluted 1:10 in serum-free RPMI 1640 (Thermo Fisher Scientific, Waltham, MA, USA), heat-inactivated at 56 °C for 30 min to remove complement activity, and mixed 1:1 with recombinant rASFV-Gluc/EGFP (10^4^ TCID_50_/mL). The virus and serum mixtures were incubated at 37 °C for 1.5 h to allow antibody–virus binding, and then 100 μL was added to PAM-containing wells. At 48 h post-infection (hpi), viral replication was assessed by two complementary methods. First, EGFP fluorescence intensity was observed under a fluorescence microscope as a qualitative indicator of infection. Second, for quantitative analysis, 30 cell culture supernatants were transferred to a black 96-well plate, mixed with 50 μL of Gluc substrate diluted 1:10 in assay buffer, incubated at room temperature in the dark for 5 min, and Gluc activity was measured using a multimode microplate (Thermo Fisher Scientific, Waltham, MA, USA) reader. Anti-ASFV sera served as the positive control, and SPF pig sera served as the negative control throughout the assay.

### 2.7. ADCC Measurement

HEK293T cells were seeded in 96-well plates at a density of 5 × 10^4^ cells/well and incubated with porcine serum antibodies (collected at 0 or 42 dpv) at dilutions of 1:10 and 1:100 at 37 °C for 2 h. Porcine peripheral blood mononuclear cells (PBMCs), isolated from EDTA-anticoagulated blood using a density gradient kit (Solarbio, Beijing, China), were added at 5 × 10^5^ cells/well (E:T ratio = 10:1) as effector cells. After 5 h co-culture, the supernatants were collected and lactate dehydrogenase (LDH) release was quantified using the CytoTox 96 non-radioactive assay kit (Promega, Madison, WI, USA) according to the manufacturer’s protocols. Cytotoxicity was calculated as:

$$\%\;\mathrm{Cytotoxicity}=\frac{\left(Experimental-Effector\;Spontaneous-Target\;Spontaneous\right)}{\left(Target\;Maximum-Target\;Spontaneous\right)}\times 100.$$

### 2.8. Statistical Analysis

An unpaired *t*-test was used to determine statistical significance and performed in GraphPad Prism version 9.5 (GraphPad Software, San Diego, CA, USA). The data were presented as the mean ± SD of at least three independent experiments. *p* < 0.05 was considered statistically significant (* *p* < 0.05, ** *p* < 0.01, *** *p* < 0.001).

## 3. Results

### 3.1. In Silico Prediction of Protein

**Figure 1:**
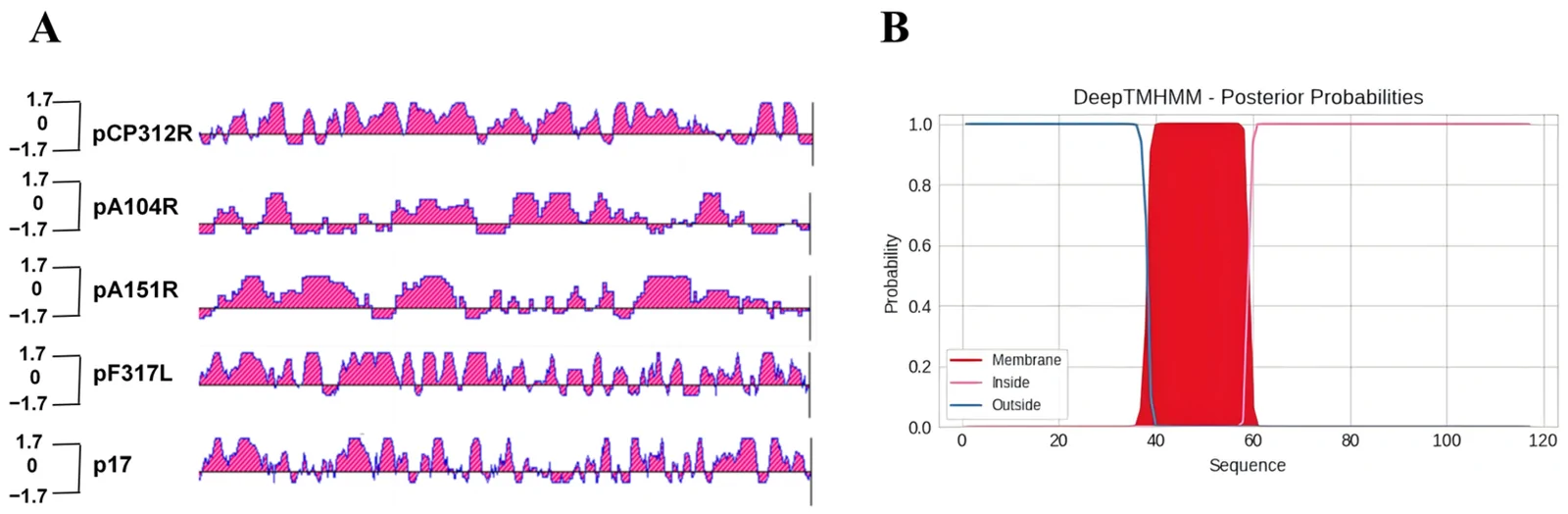
Bioinformatic characterization of candidate antigens. (**A**) Antigenic index profiles of pCP312R, pA104R, pA151R, pF317L, and p17 were analyzed using DNASTAR, highlighting potential epitopes. (**B**) Transmembrane domains on the ASFV p17 predicted by DeepTMHMM.

To evaluate the potential of the five candidate antigens as protective antigens, this study performed antigenicity index prediction using the DNASTAR software (version7.1) for pCP312R, pA151R, pA104R, p17, and pF317L. The results showed that all five proteins contain multiple highly antigenic regions at specific amino acid positions, suggesting these regions may serve as potential antigenic epitopes (Figure 1A). Furthermore, these proteins were analyzed using DeepTMHMM (https://dtu.biolib.com/DeepTMHMM, version 2.0, accessed on 20 December 2025), a deep learning-based transmembrane domain prediction tool. The analysis revealed that p17 contains a transmembrane region spanning amino acid residues 40 to 60, whereas the other four proteins lack transmembrane domains (Figure 1B).

### 3.2. Construction and Verification of Recombinant Plasmids

**Figure 2:**
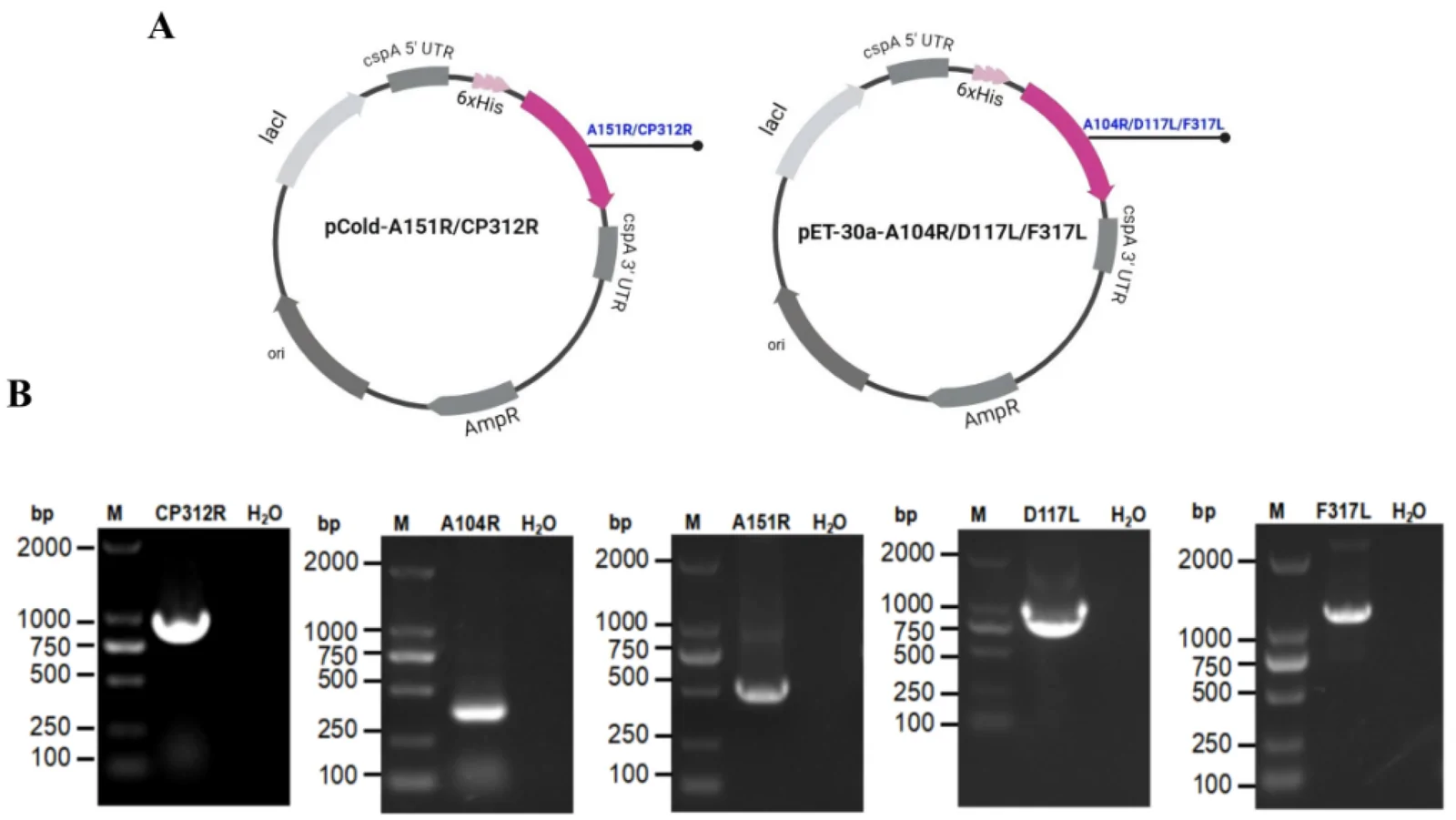
Characterization of the recombinant proteins expressed in *E. coli*. (**A**) Plasmid maps of pCold and pET30a expression constructs. (**B**) PCR identification.

The *A151R*, *CP312R*, *A104R*, *D117L-ferritin*, and *F317L* genes were codon-optimized, synthesized, and individually cloned into either pCold-I or pET-30a, resulting in recombinant plasmids designated as pCold-A151R, pCold-CP312R, pET30a-A104R, pET30a-D117L-*ferritin*, and pET30a-F317L, respectively (Figure 2A). To verify successful construction of the recombinant plasmids, PCR amplification was performed, and agarose gel electrophoresis revealed distinct bands of the expected sizes (Figure 2B). All recombinant plasmids were confirmed by Sanger sequencing.

### 3.3. Verification of Recombinant Protein Expression

**Figure 3:**
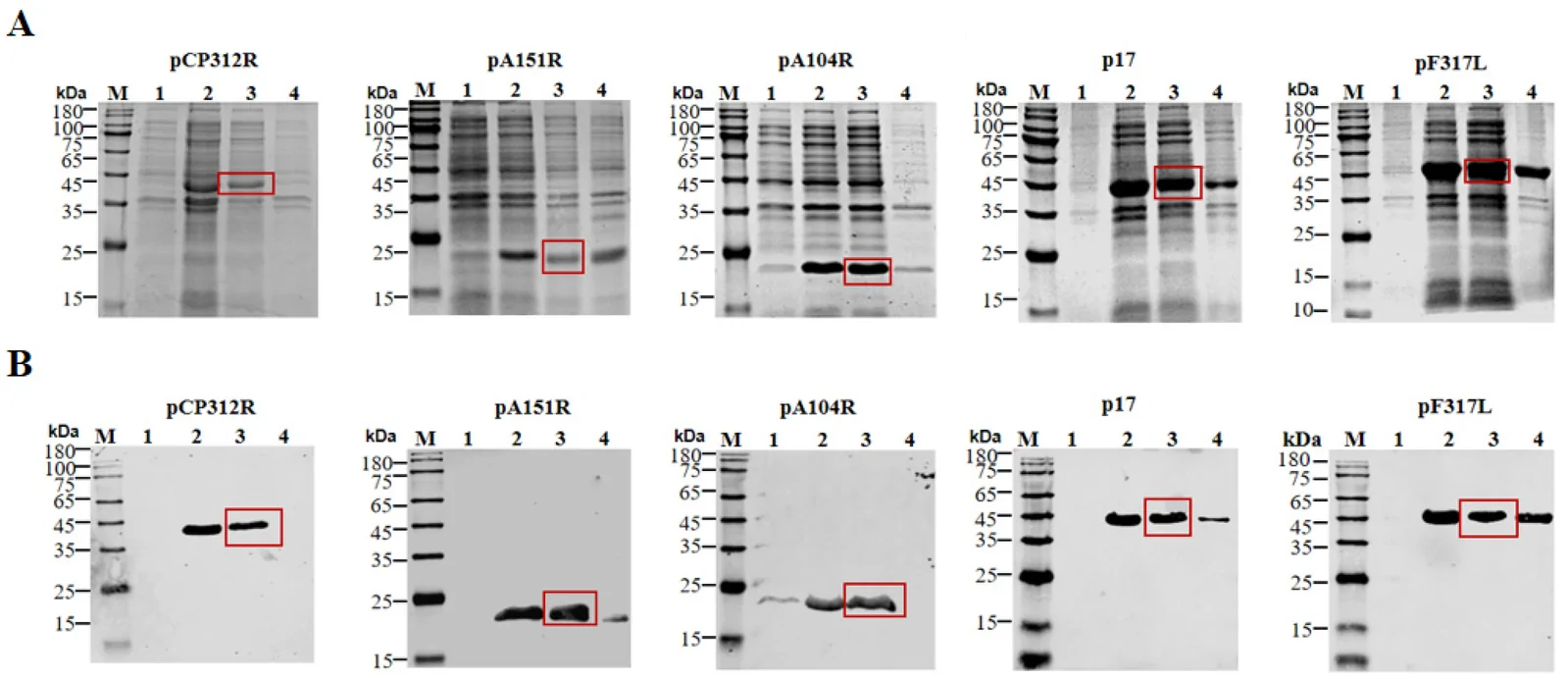
Analysis of recombinant protein expression in *E. coli*. (**A**) Protein expression analysis by Coomassie brilliant blue staining. Lane 1: Control (IPTG); Lane 2: Total lysates of IPTG-induced bacteria; Lane 3: Supernatants (soluble fraction) of induced lysate; Lane 4: Pellets (insoluble fraction) of induced lysates. (**B**) Detection of recombinant protein expression using the anti-His antibody.

SDS-PAGE analysis of soluble fractions showed that IPTG-induced BL21 (DE3) cells expressing pCP312R, pA151R, pA104R, p17-ferritin, or pF317L produced distinct protein bands at the expected sizes, while uninduced controls did not (Figure 3A). Western blotting confirmed that all recombinant proteins were specifically detected with anti-His antibody, indicating soluble expression in *E. coli* (Figure 3B).

### 3.4. Purification and Quality Assessment

**Figure 4:**
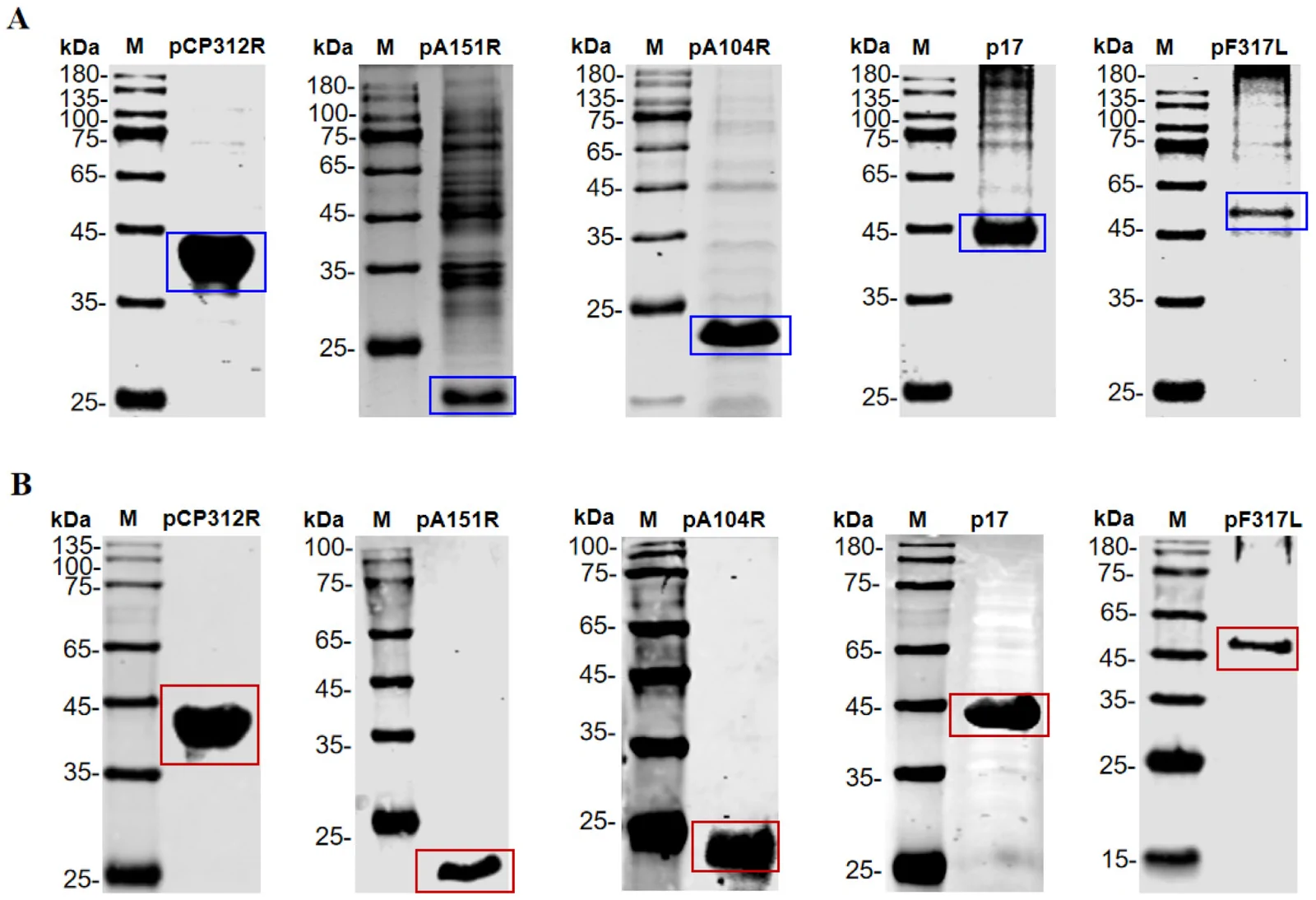
Analysis of purified recombinant proteins. (**A**) Assessment by Coomassie brilliant blue staining. (**B**) Detection by Western blotting using the anti-His antibody.

Following purification using gravity-flow columns, SDS-PAGE analysis showed a single intense band at the expected molecular weight for each protein (Figure 4A), with no detectable contaminating bands. Western blotting further confirmed that the purified proteins were efficiently recognized by an anti-His tag antibody (Figure 4B), demonstrating successful and high-efficiency purification of the target proteins.

### 3.5. Preparation and Identification of Porcine Antibodies Against ASFV Proteins

**Figure 5:**
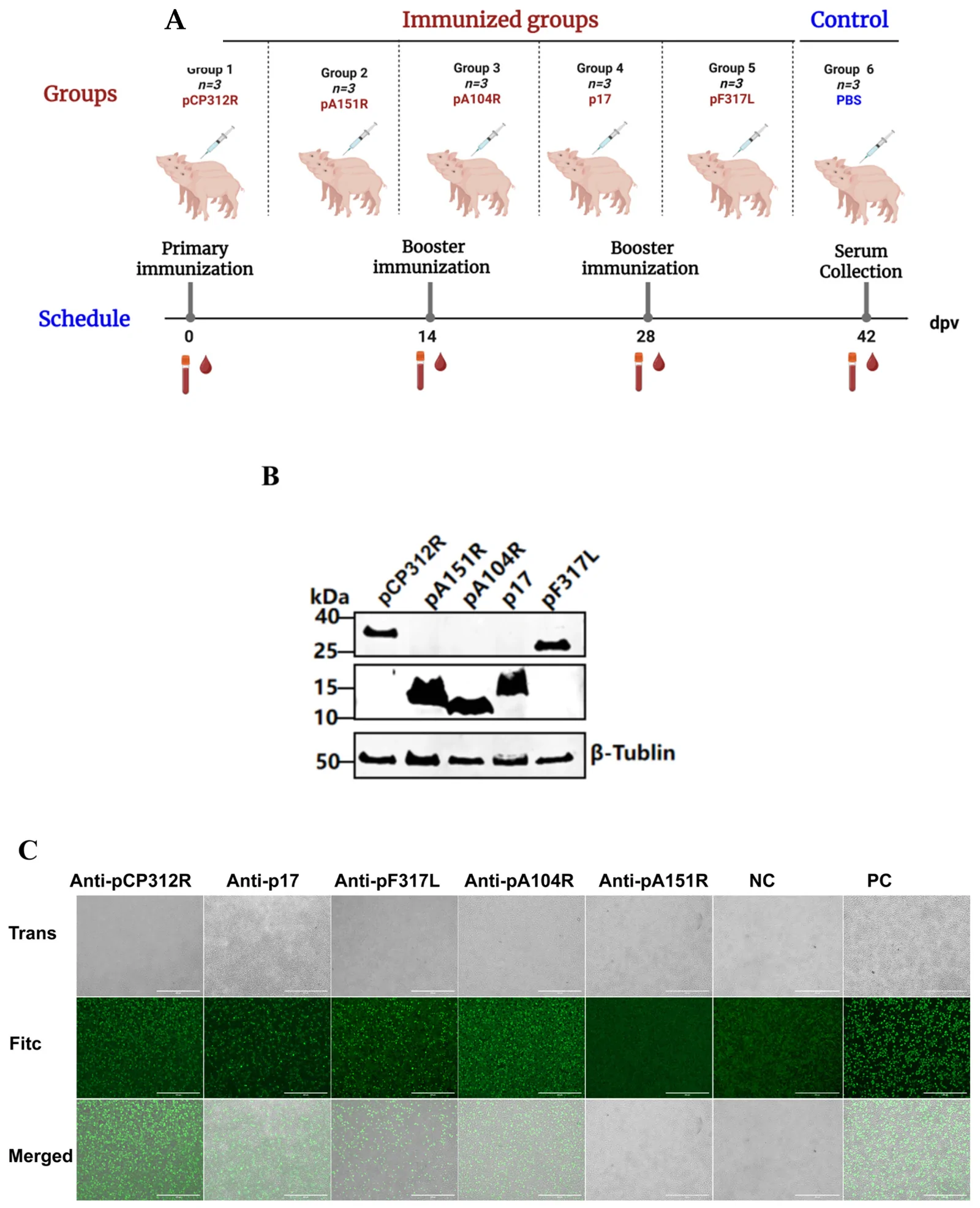
Preparation and identification of porcine antibodies against the ASFV candidate antigens. (**A**) Pig immunization schedule. Created in BioRender. Li, L. (2026) https://BioRender.com/atc01gd (accessec on 12 June 2026). (**B**) Western blotting validation of eukaryotic expression plasmids. (**C**) Detection of antigen-specific antibodies in the sera from the immunized pigs at 42 dpi by IFA. PC: Anti-ASFV sera from the pigs immunized with ASFV-SY18-ΔCD2v/UK. NC: Sera from SPF pigs. Scale bar, 400 μm.

To evaluate the immunogenicity of five candidate antigens in pigs, serum samples were collected at the indicated time points post-immunization (Figure 5A) and analyzed for antigen-specific antibodies by IFA. Western blotting confirmed that all five eukaryotic expression plasmids encoding the respective antigens were efficiently expressed in HEK293T cells (Figure 5B). The IFA results showed that, at a 1:10 dilution, the sera collected at 42 dpi produced strong green, fluorescent signals in HEK293T cells expressing pCP312R, pA104R, p17, or pF317L (Figure 5C).

### 3.6. Anti-p17 Antibodies Inhibit ASFV Replication in PAMs

**Figure 6:**
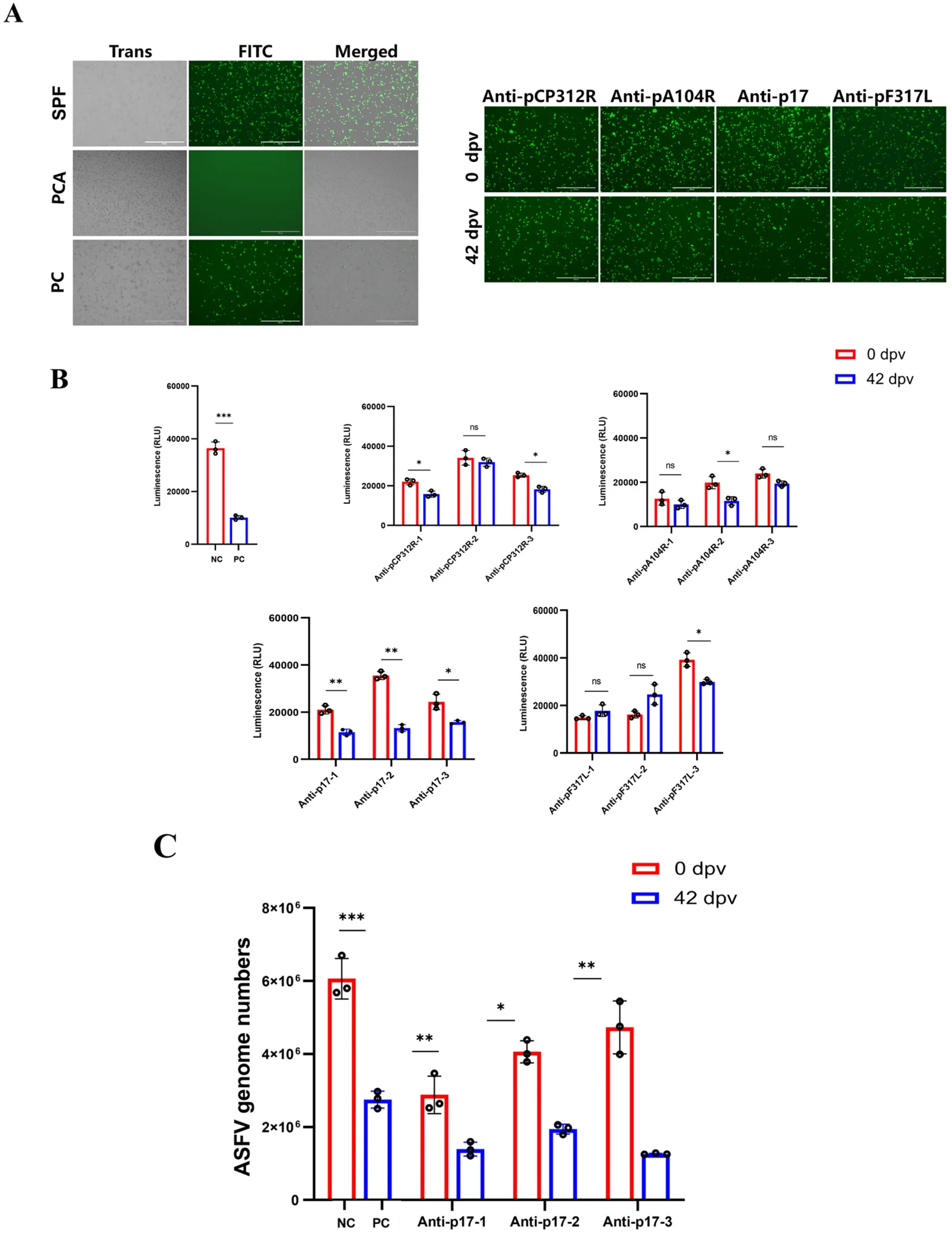
Evaluation of the immunogenicity of the recombinant p17 and the antiviral activity of anti-p17 antibodies. (**A**) EGFP expression in PAMs infected with rASFV-Gluc/EGFP under fluorescence microscope (scale bar, 400 μm). (**B**) Effects of anti-p17 sera on ASFV replication assessed by Gluc activity. (**C**) Anti-p17 sera reduced ASFV viral load as measured by qPCR. NC (negative control): SPF pig serum as negative control; PC (positive control): anti-ASFV sera from the pigs immunized with ASFV-SY18-ΔCD2v/UK. ns, not significant; * *p* < 0.05; ** *p* < 0.01; *** *p* < 0.001.

To further assess whether pCP312R, pA104R, p17, and pF317L are potentially protective antigens against ASF, we performed in vitro viral replication inhibition assays using immune sera and the recombinant virus rASFV-Gluc/EGFP. This virus stably expresses EGFP and Gluc upon infection of PAMs, enabling quantitative monitoring of viral replication [35]. Fluorescence microscopy showed weaker EGFP signal in PAMs treated with the anti-p17 sera at 42 dpi than that with the sera at 0 dpi. No inhibition was seen with anti-pCP312R, -pA104R, or -pF317L sera (Figure 6A). Gluc activity was reduced in PAMs treated with anti-p17 sera at 42 dpi compared with the sera at 0 dpi. In contrast, although the pCP312R, pA104R, and pF317L immunized groups showed a decreasing trend in some individual samples, the overall differences were not statistically significant and lacked consistency (Figure 6B). qPCR revealed that anti-p17 sera at 42 dpi significantly reduced viral genome copies in PAMs compared with those at 0 dpi (Figure 6C), confirming that anti-p17 antibodies inhibit ASFV replication in vitro. Thus, among the four candidates, only p17 is the most promising protective antigen.

### 3.7. ADCC

**Figure 7:**
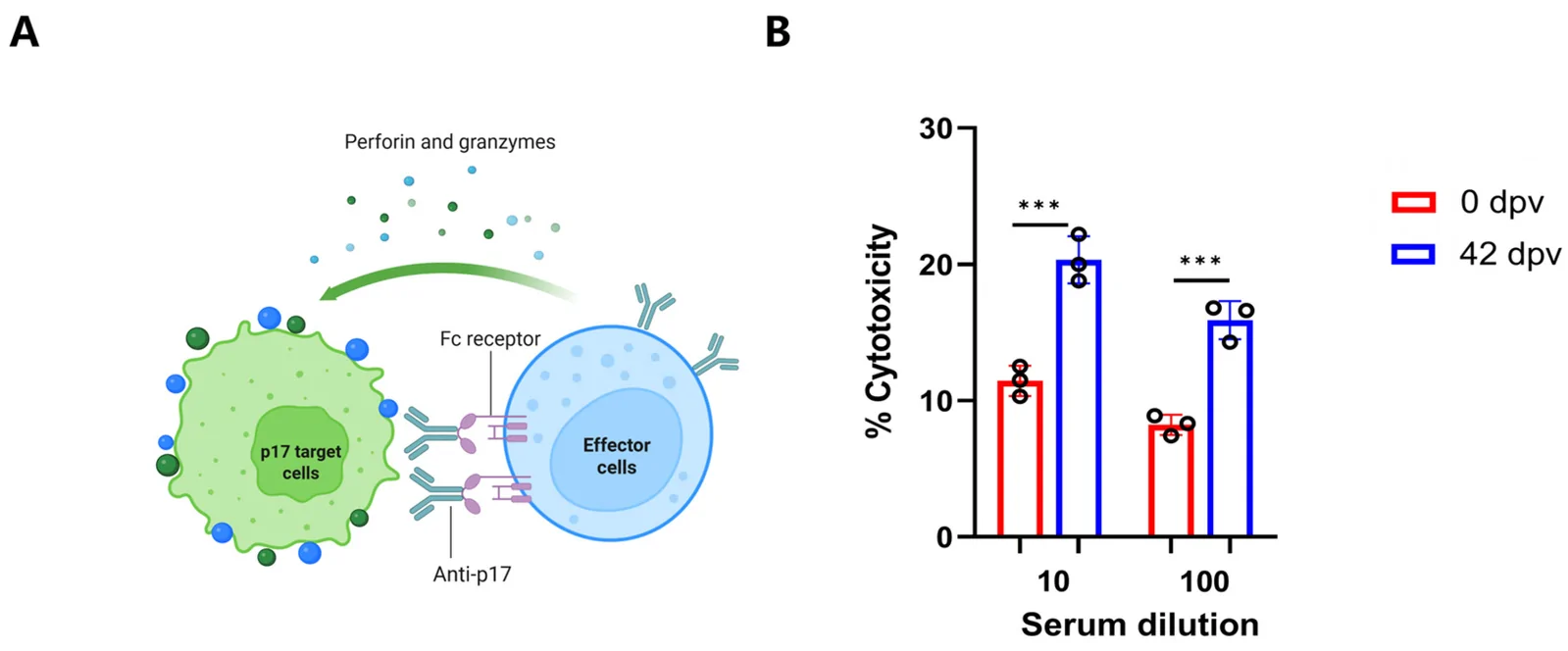
Anti-p17 antibodies induce ADCC. (**A**) Schematic illustration of the ADCC mechanism. Anti-p17 antibodies bind to p17-expressing target cells via their Fab regions, while their Fc regions engage Fc receptors on effector cells triggering degranulation and release of perforin and granzymes, leading to target cell lysis. Created in BioRender. Li, L. (2026) https://BioRender.com/t2vnq8c (accessed on 12 June 2026). (**B**) LDH release induced by anti-p17 antibodies. Statistical significance: *** *p* < 0.0001.

ADCC activity requires the presence of conformationally intact antigen on the surface of target cells for recognition by specific antibodies. In this study, HEK293T cells stably expressing the ASFV p17 protein were used as target cells to evaluate the functional activity of anti-p17 antibodies. The validity of this membrane expression system has been confirmed in our previous study [38]. In the ADCC assay, these target cells were incubated with porcine sera collected at 0 or 42 dpi, followed by the addition of PBMCs from naïve pigs as effector cells (Figure 7A). The results showed that the sera collected at 42 dpi induced higher cytotoxicity compared with the sera collected at 0 dpi, indicating that anti-p17 antibodies mediated ADCC activity (Figure 7B).

## 4. Discussion

ASFV exhibits substantial genetic heterogeneity, frequently undergoing point mutations and homologous or non-homologous recombination events during natural transmission and cross-host circulation. This leads to pronounced diversity among circulating strains in terms of whole-genome architecture, virulence phenotypes, and the composition of key antigenic epitopes [39,40,41,42]. Such complex genetic diversification not only reshapes viral pathogenesis and host adaptability, but also significantly impairs the host immune system’s ability to recognize conserved antigenic determinants, thereby limiting the induction of broad-spectrum protective immunity, which represents a major bottleneck in current ASF vaccine development [43]. In this context, the systematic identification of core structural proteins that are functionally conserved across diverse ASFV lineages, structurally stable, and highly immunogenic, holds great promise for enabling the rational design of next-generation subunit or chimeric vaccines capable of eliciting cross-strain protective immunity. In this study, five ASFV candidate antigens were selected based on the literature review and successfully expressed and purified as high-purity recombinant proteins in a prokaryotic system. Pig immunization experiment revealed that, with the exception of pA151R, all four other antigens elicited robust antigen-specific antibodies, demonstrating strong immunogenicity. Notably, in vitro functional evaluation revealed that only the serum from the p17-ferritin-immunized pigs could significantly suppress ASFV replication in PAMs. This suppression was validated through dual approaches using both the rASFV-Gluc/EGFP fluorescent reporter system and qPCR.

The failure to detect anti-pA151R antibodies likely stems from its protein properties and the immunization approach used. Although our prokaryotic expression system efficiently produced soluble protein, it may not fully recapitulate the native conformation. Thus, the current results reflect the performance of pA151R under a prokaryotic subunit immunization strategy and do not rule out its immunogenicity during natural infection. Future delivery via eukaryotic vectors or virus-like particles, combined with potent adjuvants, might better present conformational epitopes and elicit effective humoral responses [44,45]. Although pCP312R, pA104R, and pF317L induced antibodies, they showed no consistent or significant antiviral activity in replication inhibition assays. While some individuals exhibited modest inhibition, responses were overall unstable and highly variable. Some possible reasons include: First, the induced antibodies may recognize non-functional epitopes or lack sufficient affinity to block critical steps in the viral life cycle. Second, pA104R can be shielded by the virus factory and other virion layers including the core shell, inner membrane and capsid. Progressive virus morphogenesis means that at different stages pA104R may be shielded by different layers [46]. Third, the ASFV genome is highly complex and may contain redundant functional mechanisms, limiting the efficacy of targeting a single protein [47]. Therefore, antigen selection for ASF vaccines should move beyond simply assessing whether an antigen can induce antibodies and instead focus on its capacity to elicit antibodies with demonstrable antiviral functions. To verify the feasibility of p17 as an antibody effector function target, this study utilized HEK293T cells with stable p17 protein expression on the cell membrane as target cells for in vitro ADCC experiments, combined with porcine PBMCs as effector cells. The experimental results showed that anti-p17 antibodies effectively mediated specific killings of p17-expressing cells by PBMCs. Combining the dual functions of virus replication inhibition and ADCC activity discovered in p17 antibodies in this study, p17 may possess unique advantages as a vaccine antigen. Antibodies may interfere with related functions of p17 during virus assembly or maturation processes by binding to p17, thereby suppressing viral replication. In summary, p17 emerges as a strong candidate for subunit vaccines against ASF because it is highly conserved across diverse ASFV strains, including genotypes I and II as well as recombinant variants, plays an essential role in the viral life cycle, and can induce multifunctional antibodies that both suppress viral replication and mediate cytotoxic effector functions [31]. Despite the statistically significant reduction in viral replication and the induction of ADCC by p17-specific antibodies observed in the present study, the modest magnitude of the antiviral effect suggests that humoral immune mechanisms alone may be insufficient to provide sterilizing protection against highly virulent ASFV challenge. This is consistent with growing evidence that cellular immunity, rather than antibody-mediated effects, has a principle correlation with protection against ASFV. Marín-Moraleda et al. demonstrated that distinct cytotoxic cell subsets differentiate protective from non-protective immunity [48], while Lotonin et al. identified early and robust T-cell responses, not antibody titers, as tightly linked to survival [49]. Similarly, Kumar et al. showed that protection conferred by a live-vectored antigen cocktail vaccine correlated with potent T-cell responses against multiple viral antigens [50]. Future studies should focus on three priority areas: in vivo validation in pig challenge models of the ability of p17-induced antibodies to suppress viral replication; precise mapping of linear and conformational B-cell epitopes through peptide-scanning or structure-guided mutagenesis; and rational vaccine formulation strategies such as fusion with T-helper epitopes, encapsulation in nanoparticle delivery systems, or co-administration with Th1-polarizing adjuvants to promote robust T-cell help and IgG2/IgG3 subclass switching, which are linked to enhanced antibody effector functions in swine [51,52,53]. Collectively, these features make p17 a promising antigen to be included in multivalent subunit vaccines against ASF.

## 5. Conclusions

This study identified the p17 protein of ASFV as a promising protective antigen, against which specific antibodies exert dual antiviral functions by significantly inhibiting ASFV replication in PAMs and mediating potent ADCC in vitro.

## Data Availability

The original contributions presented in this study are included in the article. Further inquiries can be directed to the corresponding authors.
